# Value of the systemic immunoinflammatory index, nutritional risk index, and triglyceride-glucose index in predicting the condition and prognosis of patients with hypertriglyceridemia-associated acute pancreatitis

**DOI:** 10.3389/fnut.2025.1523046

**Published:** 2025-01-30

**Authors:** Yuan Zhen Wang, Ya Ling Yun, Ting Ye, Wen Tun Yao, Yu Feng Guo, Li Ya Huang

**Affiliations:** Gastroenterology Department, General Hospital of Ningxia Medical University, Yinchuan, China

**Keywords:** acute pancreatitis, hypertriglyceridemia, systemic immunoinflammatory index, nutritional risk index, triglyceride glucose index

## Abstract

**Objective:**

The study aimed to investigate the function and prognosis of pancreatitis in patients with hypertriglyceridemia-associated acute pancreatitis (HTGAP), as assessed by the systemic immunoinflammatory index (SII), nutritional risk index (NRI), and triglyceride-glucose index (TyG).

**Methods:**

A total of 300 patients with HTGAP who were admitted to the General Hospital of Ningxia Medical University from January 2022 to June 2023 were selected. These patients were divided into three groups based on the severity of their condition: the mild acute pancreatitis (MAP) group, the moderate-to-severe acute pancreatitis (MSAP) group, and the severe acute pancreatitis (SAP) group. The SII, NRI, and TyG index in the three groups were recorded and compared. The value of these indices in predicting the occurrence of HTGAP was analyzed using a receiver operating characteristic (ROC) curve.

**Results:**

The SII and TyG index values in the SAP group (3259.4 ± 2795.8, 4.5 ± 1.1) were higher than those in the MSAP group (2563.7 ± 1614.1, 4.3 ± 0.8) and MAP group (1991.1 ± 1566.8, 4.1 ± 0.8), and the difference was statistically significant (*p* < 0.005). The ROC curve analysis showed that the AUC value of the combined SII, NRI, and TyG index for predicting SAP occurrence was 0.705 (95%CI:0.632 ~ 0.778).

**Conclusion:**

The SII, NRI, and TyG index are related to the severity of HTGAP, and a combination of the three can better predict the occurrence of SAP.

## 1 Introduction

Acute pancreatitis (AP) is one of the most common clinical emergencies, associated with high morbidity. In recent years, hypertriglyceridemia has become the second most common cause of AP (1). Compared with biliary pancreatitis, patients with hypertriglyceridemia-associated acute pancreatitis (HTGAP) tend to be younger, have more severe pancreatitis, experience more complications, and have higher morbidity (2). Research has found that approximately 20% of patients can develop severe acute pancreatitis (SAP), which has a high fatality rate and seriously threatens patients’ lives (3). The systemic immunoinflammatory index (SII) is a new inflammatory marker that can more stably reflect the inflammatory state of the body and has high value in assessing the severity and prognosis of digestive diseases (4). The nutritional risk index (NRI), a commonly used tool for the clinical assessment of nutritional status, is simple, comprehensive, and economical and is of great value for the assessment of the nutritional status of critically ill patients (5). The triglyceride-glucose (TyG) index is a marker that reflects the metabolic status of the body. Inflammation and abnormal metabolism can trigger and aggravate AP, and it is speculated that TyG is associated with the occurrence and development of AP (6). However, there have been few reports on the application value of the SII, NRI, and TyG index in HTGAP. Therefore, by analyzing the SII, NRI, and TyG index in patients with HTGAP, this study explored their value in predicting the occurrence of SAP, aiming to assist in the diagnosis and treatment of SAP.

## 2 Materials and methods

### 2.1 Research participants

A total of 300 patients with HTGAP who were admitted to our hospital from January 2021 to January 2023 were included in the study.

#### 2.1.1 Inclusion criteria

The inclusion criteria were in accordance with the Chinese Guidelines for Diagnosis and Treatment of Acute Pancreatitis (2019) (7), and those who could cooperate and complete the research were included.

#### 2.1.2 Exclusion criteria

The exclusion criteria included the following: A: Patients with severe cardiopulmonary disease, severe liver and kidney disease, and cerebrovascular disease. B: Patients with a history of pancreatic cancer or pancreatic surgery. C: Pregnant or lactating women.

This study was approved by the Ethics Committee of the hospital, and informed consent was obtained from the patients.

### 2.2 Clinical data

According to the criteria outlined in the Chinese Guidelines for Diagnosis and Treatment of Acute Pancreatitis (2019), the patients with AP were grouped based on their clinical manifestations and severity. A total of 203 cases of mild acute pancreatitis (MAP) were reported, with no organ failure or local complications. Forty-seven patients with moderate-to-severe acute pancreatitis (MSAP) had no local complications and experienced single organ failure for less than 48 h. A total of 71 cases of SAP experienced persistent organ failure lasting more than 48 h, with local complications. Information such as gender, age, body mass index, etiology, past medical history, and laboratory indicators were collected.

### 2.3 Observation of the indicators

The NRI on the day of HTGAP onset and admission was recorded as follows: 1.489*serum albumin (g/L) + 0.417* [actual body mass/ideal body mass (kg) *100]. The SII was recorded as follows: Platelet count *neutrophil count/lymphocyte count. The TyG index was calculated as follows: Ln[fasting triglyceride (mg/dL) *fasting blood glucose (mg/ dL)/2].

Ideal body mass (kg) = body height (cm)−105, the value calculated by the ideal body mass calculation formula, which belongs to the ideal body mass within the range of ±5%.

### 2.4 Ethics approval and consent to participate

The study was approved by the Ethics Committee, General Hospital of Ningxia Medical University (KYLL-2023-0021). The study procedures were performed in accordance with the Declaration of Helsinki’s ethical principles for medical research involving human participants. Informed consent was obtained from all patients included in the study.

### 2.5 Statistical methods

The SPSS22.0 statistical software was used for the statistical analysis of the data. The measurement data with a normal distribution were presented as (x_ ± s). One-way analysis of variance was used for comparisons among the groups, and the LSD-t test was used for pairwise comparisons. The measurement data with a non-normal distribution were expressed as [M (P25 ~ P75)], and the non-parametric Kruskal–Wallis H test was used for comparisons between the groups. The statistical data were all expressed as the number of cases (percentage), and the χ2 test was used for comparisons between the groups. The receiver operating characteristic curve (ROC) was used and the area under the curve (AUC) was calculated as an objective index to evaluate the performance. The comparison of AUC was performed by Z-test. Using the non-SAP group and SAP group as variables, the values of the NRI, SII, and TyG index in predicting SAP occurrence were analyzed. A *p*-value of <0.05 was considered statistically significant.

## 3 Results

### 3.1 Comparison of the clinical baseline data and laboratory indicators in each group

There were statistically significant differences in albumin, blood glucose, neutrophil relative value, blood creatinine, white blood cell count, lactate dehydrogenase, blood calcium, blood potassium, and length of stay between the SAP group, MSAP group, and MAP group (*p* < 0.05, Table 1).

**Table 1:** Comparison of the clinical baseline data and laboratory indicators in each group.

Content	MAP (*N* = 203)	MSAP (*N* = 47)	SAP (*N* = 71)	χ^2^/F	*p*
Smoking [*n*(%)]				2.773	0.250
No	109 (53.7%)	22 (46.8%)	44 (62%)		
Yes	94 (46.3%)	25 (53.2%)	27 (38%)		
Alcohol consumption [*n*(%)]				2.937	0.230
No	119 (58.9%)	22 (46.8%)	44 (62%)		
Yes	83 (41.1%)	25 (53.2%)	27 (38%)		
Hypertension [*n*(%)]				0.595	0.743
No	183 (90.1%)	41 (87.2%)	65 (91.5%)		
Yes	20 (9.9%)	6 (12.8%)	6 (8.5%)		
Diabetes [*n*(%)]				2.412	0.299
No	143 (70.4%)	31 (66%)	43 (60.6%)		
Yes	60 (29.6%)	16 (34%)	28 (39.4%)		
Fatty Liver [*n*(%)]				2.990	0.224
No	56 (27.6%)	8 (17%)	22 (31%)		
Yes	147 (72.4%)	39 (83%)	49 (69%)		
BMI(Mean ± SD)	26.9 ± 4.1	27.1 ± 2.8	27.4 ± 5.1	0.373	0.689
Albumin(Mean ± SD)	43.5 ± 5.0	42.5 ± 5.3	39.2 ± 6.4	17.253	<0.001
PLT(Mean ± SD)	242.2 ± 58.5	256.1 ± 55.3	243.5 ± 71.4	0.998	0.370
Glucose(Mean ± SD)	9.5 ± 4.1	10.9 ± 5.3	14.2 ± 8.7	18.114	<0.001
TC(Mean ± SD)	16.2 ± 10.9	18.4 ± 14.0	19.0 ± 15.8	1.578	0.208
LYMP(Mean ± SD)	4.6 ± 39.8	3.4 ± 12.0	1.6 ± 2.3	0.227	0.797
NEUT(Mean ± SD)	10.9 ± 4.5	13.3 ± 4.2	13.6 ± 4.2	12.098	<0.001
CREA(Mean ± SD)	63.0 ± 13.7	61.1 ± 14.4	91.2 ± 75.2	16.299	<0.001
Length of stay(Mean ± SD)	7.1 ± 3.5	11.0 ± 3.9	11.9 ± 7.6	32.371	<0.001
WBC(Mean ± SD)	13.4 ± 4.5	15.7 ± 4.5	15.9 ± 4.7	10.468	<0.001
UA(Mean ± SD)	415.8 ± 116.1	408.5 ± 119.4	444.2 ± 187.4	1.391	0.250
LDH(Mean ± SD)	539.0 ± 195.9	718.2 ± 299.3	1164.4 ± 1005.1	39.638	<0.001
TG(Mean ± SD)	7.6 ± 2.7	8.5 ± 3.1	7.6 ± 3.0	1.877	0.155
CA(Mean ± SD)	2.3 ± 0.2	2.2 ± 0.2	2.0 ± 0.3	37.382	<0.001
K (Mean ± SD)	4.2 ± 0.4	4.2 ± 0.5	4.5 ± 0.9	6.446	0.002

### 3.2 Comparison of the SII, NRI, and TyG index in the patients with HTGAP of different severity

The NRI value of the SAP group was lower than that of the MSAP group and MAP group (*p* < 0.005), and the SII and TyG index values of the SAP group were higher than those of the MSAP group and MAP group, with statistical significance (*p* < 0.005, Table 2).

**Table 2:** Comparison of the NRI, SII, and TyG index values in the patients with AP of different severity.

Group	*n*	NRI	SII	TyG index
MAP	203	114.4 ± 11.4	1991.1 ± 1566.8	4.1 ± 0.8
MSAP	47	113.6 ± 10.4	2563.7 ± 1614.1	4.3 ± 0.8
SAP	71	109.1 ± 13.6	3259.4 ± 2795.8	4.5 ± 1.1
F	-	5.363	11.896	5.694
*p*	-	0.005	<0.001	0.004

### 3.3 Value of SAP occurrence predicted by the SII, NRI, and TyG index

**Figure 1:**
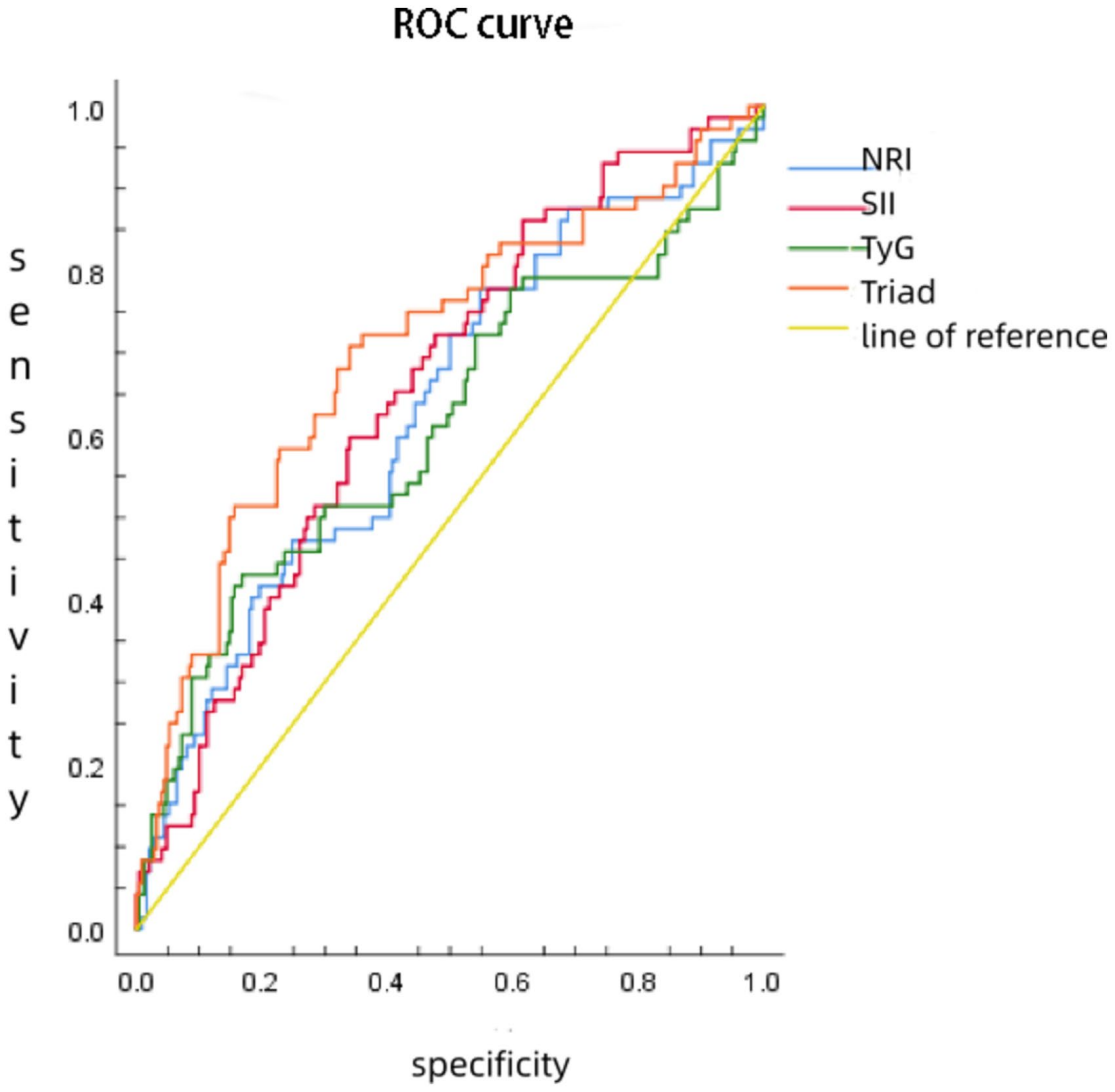
ROC curve of the NRI, SII, and TyG index predicting SAP occurrence.

The best truncation values of the SII, NRI, and TyG index for SAP prediction were 2318.41, 115.53, and 4.832, respectively. The AUC value of the combination of the SII, NRI, and TyG index was higher than that of the indexes when used individually in predicting SAP occurrence (all *p* < 0.05), and its sensitivity and specificity were 70.8 and 66% (Table 3; Figure 1).

**Table 3:** Value of SAP occurrence predicted by the NRI, SII, and TyG index.

Gourp	Cutoff	AUC (95% CI)	Sensitivity	Specificity	Positive predictive value	Negative predictive value
NRI	115.53	0.633(0.558–0.708)	0.778	0.452	0.290	0.876
SII	2318.41	0.655(0.586–0.725)	0.597	0.660	0.336	0.851
TyG Index	4.823	0.612(0.530–0.694)	0.431	0.832	0.425	0.835
Triad	-	0.705(0.632–0.778)	0.708	0.660	0.375	0.887

## 4 Discussion

SAP is one of the common acute and severe conditions in digestive diseases, and the risk of death can increase if treatment is not administered promptly. Early recognition and prognostic prediction of SAP are of great significance for early clinical treatment and for improving the disease outcomes. Multiple studies have shown (8) that, compared to AP caused by other factors, patients with HTGAP are mostly young, experience faster and more severe disease progression, have a poor prognosis, and have a high recurrence rate. Due to the special clinical manifestations of HTGAP, clinicians are required to accurately and efficiently assess the severity of the disease and provide targeted monitoring measures and treatment plans for potentially severe patients as soon as possible. As a screening tool for monitoring nutritional status, the NRI has the advantage of being objective, practical, and easy to use and has good application value in digestive tract diseases (9). As an evaluation index reflecting the immune and inflammatory states of the body, the SII has been widely used in clinical practice due to its simple calculation, low cost, and high feasibility and is of great value in the assessment and prognosis of pancreatic cancer (10). Studies have shown that the SII can not only be used as a prognostic factor for malignant tumors but is also related to the occurrence, development, severity, and prognosis of digestive system diseases (11). As a marker for assessing insulin resistance, the TyG index is convenient, reliable, and efficient and is widely used in the diagnosis and treatment of coronary heart disease. It is of great significance in predicting the occurrence, severity, and poor prognosis of coronary heart disease (12). The TyG index is calculated using fasting blood glucose and triacylglycerol levels. Oxidative stress induced by hyperglycemia can promote the inflammatory reaction process in the pancreas and is independently correlated with the severity and mortality of AP (13). An increase in triglyceride levels can aggravate pancreatic ischemia and hypoxia, disrupt microcirculation, and induce a systemic proinflammatory reaction, which is associated with AP complications (14). Therefore, it is speculated that an increase in the TyG index value may be related to a greater tendency for severe AP.

The results of this study showed that the NRI level of the patients with HTGAP gradually decreased with the severity of the disease and that the NRI level of the SAP group was lower than that of the MSAP group and MAP group. Meanwhile, the SII and TyG index levels of the patients with HTGAP gradually increased with the severity of the disease, and the SII and TyG index levels of the SAP group were higher than those of the MSAP group and MAP group, indicating a low NRI level. The increase in the SII and TyG index promoted and was closely related to the severity of HTGAP. The aggravation of the disease in patients with HTGAP may be related to the decrease in immune function and the enhancement of the inflammatory response caused by poor nutrient intake. Liu (15) retrospectively analyzed 101 patients with AP, and the results showed that the SII was related to the severity of AP and could be used as an effective indicator to assess its disease progression and prognosis. Li (16) observed that the TyG index integrated information from both glucose and lipid metabolism and that its increase was related to the onset and severity of AP, making it an independent risk factor for SAP.

The ROC curve analysis in this study showed that the SII, NRI, and TyG index had certain value in predicting the occurrence of severe HTGAP, and the combination of the SII, NRI, and TyG index demonstrated clear advantages over the indexes when used individually in predicting the occurrence of SAP. This may be due to the combined effects of the nutritional index (NRI), inflammatory index (SII), and TyG index, which aggravate oxidative stress, inflammatory response, and disturbances in glucose and lipid metabolism, promote damage to the pancreas and other organs, and ultimately lead to multiple organ failure and death in patients with SAP. Beata (17) believed that reduction in the NRI is related to the risk of death in patients with SAP and that the NRI is of certain value in the nutritional risk assessment and prognosis for patients with SAP. Murat (18) showed that the SII value was significantly increased in patients with SAP and was related to the degree of disease progression, which may be a biological indicator for predicting poor prognosis for patients with SAP. Previous studies have also found that the TyG index value in the SAP group was higher than that in the non-SAP group. The TyG index is an independent prognostic factor for SAP and can be used as a simple indicator for predicting the prognosis of SAP (19).

In summary, the NRI, SII, and TyG index are related to the severity of disease in patients with HTGAP, and the combination of the three indexes has significant value in predicting the occurrence and prognosis of SAP. It is expected to become a new combined marker for predicting the prognosis of SAP. However, this was a retrospective, single-center study with a small sample size, which might have led to bias in the study results. Further verification through large-scale, multi-center clinical studies is needed.

## Data Availability

The original contributions presented in the study are included in the article/supplementary material, further inquiries can be directed to the corresponding author.
